# Pristimerin, a quinonemethide triterpenoid, induces apoptosis in pancreatic cancer cells through the inhibition of pro-survival Akt/NF-κB/mTOR signaling proteins and anti-apoptotic Bcl-2

**DOI:** 10.3892/ijo.2014.2325

**Published:** 2014-03-05

**Authors:** DORRAH DEEB, XIAOHUA GAO, YONG BO LIU, KIRIT PINDOLIA, SUBHASH C. GAUTAM

**Affiliations:** 1 Departments of Surgery, Henry Ford Health System, Detroit, MI 48202, USA; 2 Medical Genetics, Henry Ford Health System, Detroit, MI 48202, USA

**Keywords:** pristimerin, pancreatic cancer, apoptosis, prosurvival signaling, Bcl-2

## Abstract

Lack of effective therapeutics for pancreatic cancer at the present time underscores the dire need for safe and effective agents for the treatment of this malignancy. In the present study, we have evaluated the anticancer activity and the mechanism of action of pristimerin (PM), a quinonemethide triterpenoid, against MiaPaCa-2 and Panc-1 pancreatic ductal adenocarcinoma (PDA) cell lines. Treatment with PM inhibited the proliferation and induced apoptosis in both cell lines as characterized by the increased Annexin V-binding and cleavage of PARP-1 and procaspases -3, -8 and -9. PM also induced mitochondrial depolarization and the release of cytochrome *c* from the mitochondria. The induction of apoptosis by PM was associated with the inhibition of the pro-survival Akt, NF-κB and mTOR signaling proteins and their downstream intermediaries such as Foxo-3α and cyclin D1 (Akt); Cox-2 and VEGF (NF-κB); p-S6K1 and p-4E-BP1 (mTOR) as well as PKCɛ. Treatment with PM also inhibited the expression of anti-apoptotic Bcl-2 and survivin but not Bcl-xL. The downregulation of Bcl-2 by PM was not due to proteasomal or lysosomal proteolytic degradation of Bcl-2, since treatment with PM in the presence of proteasomal inhibitors MG132 or lactacystin (LAC) or calpain inhibitor MG101 failed to block the downregulation of Bcl-2 by PM. On the other hand, RT-PCR analysis showed the inhibition of Bcl-2 mRNA by PM in a dose-related manner, indicating that inhibition of Bcl-2 by PM is mediated through the suppression of Bcl-2 gene expression. Thus, the mechanistic understanding of the antitumor activity of pristimerin could facilitate *in vivo* efficacy studies of pristimerin for pancreatic cancer.

## Introduction

Pancreatic ductal adenocarcinoma (PDA) is the fourth leading cause of cancer-related deaths in the United States and is almost uniformly lethal with an estimated annual incidence of 43,140 new cases approximating 36,800 annual deaths and a 5-year survival rate of <5% (1–3). Late initial diagnosis, aggressive metastatic behavior and resistance to chemoradiotherapy render pancreatic cancer one of the most difficult to treat malignant diseases. Surgical resection is curative; however, nearly 80% of the patients are diagnosed with locally advanced metastatic disease, precluding surgical intervention. Gemcitabine, the current standard of care for advanced pancreatic cancer, provides only short-term symptomatic improvement with minor impact on survival (4,5). The integration of multiple modalities also has not improved survival. Thus, there is the dire need for more active agents and novel strategies for the treatment of pancreatic cancer.

Herbal remedies are used in traditional medicine to treat and prevent human diseases including cancer. Numerous plant derived flavonoids and phenolic/polyphenolic compounds with antioxidant and anti-inflammatory activities are currently used by cancer patients as dietary supplements to complement chemotherapy. In fact, isolation and identification of bioactive components from medicinal plants have led to the synthesis and development of potent anticancer drugs, including Vinca alkaloids, taxol, camptothecan, etoposide and retinoids. Triterpenoids are members of a larger family of structurally related compounds known as cyclosqualenoids that are widely distributed in nature. Pristimerin (PM) is a quinonemethide triterpenoid present in various plant species in the *Celastraceae* and *Hippocrateaceae* families (6,7). PM and related compounds have shown anti-inflammatory, antioxidant and antimalarial activities (8–10). Recent studies have shown potent antiproliferative and apoptosis-inducing activity of PM in glioma, leukemia, breast, lung and prostate cancer cell lines (11–14). Induction of apoptosis by PM involved activation of caspases, mitochondrial dysfunction, inhibition of anti-apoptotic nuclear factor-κB (NF-κB) and Akt (15–17). Recent evidence also shows participation of reactive oxygen species (ROS) in the antitumor activity of PM (18). In addition, PM also inhibited proteasome activity, tumor cell migration and angiogenesis (13,19,20).

To the best of our knowledge there has been only one published report showing the antitumor activity of PM against pancreatic cancer cells through the inhibition of cell cycle progression and induction of apoptosis (21). Since this report, there has been no additional report on the anticancer activity and mechanism(s) of action of PM in pancreatic cancer cells. In the present study, we demonstrate that PM inhibits proliferation and induces apoptosis in pancreatic cancer cells by inhibiting the pro-survival Akt, NF-κB and mTOR signaling proteins and their downstream mediators as well as anti-apoptotic Bcl-2. The inhibition of Bcl-2 by PM was not through the proteolytic degradation but resulted from the inhibition of Bcl-2 gene transcription.

## Materials and methods

### Reagents

Pristimerin was purchased from Sigma Chemicals (St. Louis, MO). Anti-caspase-3 and caspase-9 antibodies were purchased from BD Pharmingen (San Diego, CA). Anti-PARP-1, anti-Bcl-2, anti-Bcl-xL and anti-survivin antibodies were purchased from Santa Cruz Biotechnology (Santa Cruz, CA). 96 AQueous One Solution Proliferation Assay System was from Promega (Madison, WI). Annexin V-FITC apoptosis detection kit was purchased from BD Pharmingen and mitochondrial potential sensor JC-1 was obtained from Molecular Probes, Invitrogen (San Diego, CA). Caspase-3 activity kit was from Clontech Laboratories (Mountain View, CA). Stock solution of PM (100 mM) was prepared in DMSO and all test concentrations were prepared by diluting stock solution in tissue culture medium.

### Cell lines

MiaPaCa-2 and Panc-1 PDA cell lines were obtained from the American Type Culture Collection (ATCC, Rockville, MD). Both cell lines were grown in DMEM tissue culture medium (Gibco-BRL, Rockville, MD) supplemented with 10% fetal bovine serum, 1% penicillin/streptomycin, and 25 mM HEPES buffer. Cells were incubated at 37°C in a humidified atmosphere consisting of 5% CO_2_, 95% air and maintained by splitting cultures twice a week.

### Measurement of cell viability (MTS assay)

Tumor cells (1×10^4^) in 100 *μ*l of tissue culture medium were seeded into each well of a 96-well plate. After 24-h incubation to allow cells to adhere, cells were treated with PM at concentrations ranging from 0 to 5 *μ*M. Cultures were incubated for additional 72 h and cell viability was then determined by the colorimetric MTS assay using CellTiter 96 AQueous One Solution Proliferation Assay System from Promega. This assay measures the bioreduction of the tetrazolium compound MTS by intracellular dehydrogenases in the presence of electron-coupling reagent phenazine methosulfate. MTS and phenazine methosulfate were added to the culture wells, and cultures were incubated for 2 h at 37°C. The absorbance, which is directly proportional to the number of viable cells in the cultures, was measured at 490 nm using a microplate reader.

### Annexin V-FITC binding

Induction of apoptosis was assessed by the binding of Annexin V-FITC to the cells. Briefly, MiaPaCa-2 and Panc-1 cells treated with PM (0 to 5 *μ*M) for 20 h were incubated with 5 *μ*l of Annexin V-FITC and 5 *μ*l of PI for 30 min at room temperature in the dark. Stained cells were analyzed by flow cytometry.

### Western blot analysis

Cell lysates were prepared by detergent lysis [1% Triton X-100 (v/v), 10 mM Tris-HCl (pH 7.5), 5 mM EDTA, 150 mM NaCl, 10% glycerol, 2 mM sodium vanadate, 5 *μ*g/ml leupeptin, 1 *μ*g/ml aprotinin, 1 *μ*g/ml pepstatin A and 10 *μ*g/ml 4-2-aminoethyl-benzenesulfinyl fluoride]. Lysates were clarified by centrifugation at 14,000 × g for 10 min at 4°C, and protein concentrations were determined by Bradford assay. Samples (50 *μ*g) were boiled in an equal volume of sample buffer [20% glycerol, 4% SDS, 0.2% bromophenol blue, 125 mM Tris-HCl (pH 7.5), and 640 mM 2-mercaptoethanol] and separated on 10% SDS-polyacrylamide gels. Proteins resolved on the gels were transferred to nitrocellulose membranes and probed with antibodies to procaspases -3 and -9 (1:1,000), Akt, NF-κB, mTOR, Bcl-2, Bcl-xL, survivin, cytochrome *c*, Cox IV or β-actin (all at 1:500 dilution) followed by HRP-conjugated secondary antibody. Immune complexes were visualized by chemiluminescence. Protein bands were imaged and band densities analyzed using the NIH/Scion image analysis software. The protein band densities were normalized to the corresponding β-actin band densities and relative change in signal strength was calculated.

### Measurement of mitochondrial depolarization and cytocyto-chrome c release

The loss of mitochondrial potential was determined using mitochondrial potential sensor JC-1 (Molecular Probes, Invitrogen). Control or cells treated with PM (1×10^6^) were loaded with mitochondrial sensor JC-1 dye (10 *μ*g/ml) for 10 min at 22°C. Cells were then analyzed by flow cytometry. In normal cells, dye is aggregated in mitochondria, fluoresces red, and is detected in the FL2 channel. In cells with altered mitochondrial potential, the dye fails to accumulate in the mitochondria, remains as monomers in the cytoplasm, fluoresces green and is detected in the FL1 channel.

For effect on mitochondrial cytochrome *c*, mitochondrial and cytosolic fractions of control and treated cells were prepared using ApoAlert Cell Fractionation Kit (Clontech Laboratories) and were separated on a 14% SDS-PAGE gel. After transfer of proteins, membranes were probed with cytochrome *c* antibody.

### Measurement of Bcl-2 gene expression

The effect of PM on Bcl-2 gene expression was measured by analyzing Bcl-2 mRNA by RT-PCR. Total cellular RNA was extracted with TRIzol reagent (Gibco-BRL) and 1 *μ*g of RNA was reverse transcribed by oligo-dt primer and high fidelity reverse transcriptase (Boehringer, Mannheim, Germany) to generate cDNAs. cDNA was used (1 *μ*l) of as the template for polymerase chain reaction (PCR) using Bcl-2 primers: upper, 5′-CGA CTT CGC CGA GAT GTC CAG CCA G-3′; and lower, 5′-ACT TGT GGC CCA GAT AGG CAC CCA G-3′; and GAPDH primers: upper, 5′-TCC CTC AAG, ATT, GTC AGC AA-3′; and lower, 5′-AGA TCC ACA ACG GAT ACA TT-3′. The PCR reaction conditions used were: a first cycle of 10 min at 95°C, 45 sec at 65°C and 1 min at 72°C followed by 45 sec at 95°C, 45 sec at 65°C and 1 min at 72°C for 36 cycles. The PCR products were separated on 2% agarose gel and visualized by ethidium bromide staining. The size of DNA fragments amplified by Bcl-2 primers GAPDH primers was 388 and 173 bp, respectively.

### Statistical analysis

Data are presented as the means ± S.D. The differences between control and treatment groups were analyzed using Dunnett’s multiple comparison test and differences with p<0.05 were considered statistically significant.

## Results

### Pristimerin inhibits proliferation of pancreatic cancer cells

**Figure 1.**
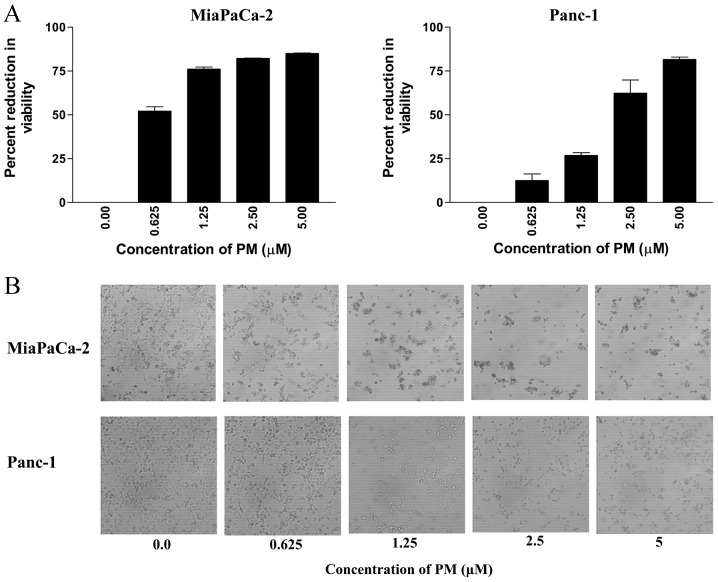
PM inhibits proliferation of pancreatic cancer cells. (A) MiaPaCa-2 or Panc-1 cells (1×10^4^) were seeded in each well of a 96-well plate. Twenty-four hours later, cells were treated with PM at concentrations ranging from 0 to 5 *μ*M for 72 h in triplicate. Cell viability was measured by MTS assay using CellTiter AQueous Assay System. ^*^p<0.05 compared to control cells. (B) Morphological changes in cell cultures visualized by light microscopy 48 h after treatment with PM. Each experiment was repeated three times.

To measure the effect of PM on proliferation of pancreatic cancer cells, MiaPaCa-2 and Panc-1 cells were treated with PM for 72 h at concentrations ranging from 0.625 to 5 *μ*M. At the end of the treatment, viability of cultures was determined by MTS assay. As shown in Fig. 1A, PM significantly reduced the proliferation of both cell lines (measured from the loss of viability of cultures) at concentrations of 0.625 to 5 *μ*M (MiaPaCa-2, 52 to 85% reduction; Panc-1, 13 to 81% reduction, p<0.05).

The antiproliferative effect of PM in MTS assay correlated with the morphological changes in cell cultures treated with PM. Microscopic examination of MiaPaCa-2 and Panc-1 cell cultures at 48 h after treatment with PM showed partial rounding of cells at 0.625 *μ*M. At higher concentrations of PM (1.25 to 5 *μ*M), there was extensive cellular detachment, rounding, shrinkage and clumping of cells in cultures of both cell lines in a dose-dependent manner (Fig. 1B). Together, MTS and morphological data indicated strong antiproliferative effect of PM on pancreatic cancer cells.

### Pristimerin induces apoptosis in pancreatic cancer cells

**Figure 2.**
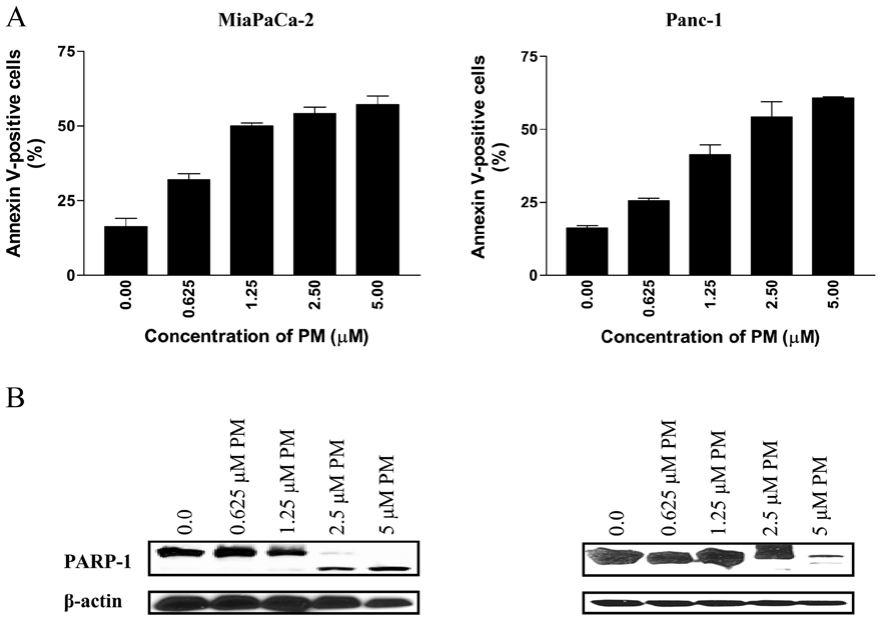
Treatment with PM induces apoptosis in pancreatic cancer cells. (A) MiaPaCa-2 and Panc-1 cells were treated with PM at 0 to 5 *μ*M for 20 h. Cells were then reacted with 5 ml of Annexin V-FITC and 5 ml PI for 30 min and percentage of Annexin V-FITC binding cells was determined by flow cytometry. (B) Cleavage of PARP-1 in MiaPaCa-2 and Panc-1 cells treated with PM (0 to 5 *μ*M) for 20 h. Similar results were obtained in three independent experiments.

To determine whether PM induces apoptosis in pancreatic cancer cells, we first measured the binding of Annexin V-FITC to MiaPaCa-2 and Panc-1 cells treated with PM by flow cytometry. As shown in Fig. 2A, treatment with PM (0.625 to 5 *μ*M) for 20 h significantly increased the percentage of Annexin V-FITC binding cells in both cell lines. In MiaPaCa-2 cells, the percentage of Annexin V-FITC-binding cells increased from 32% at 0.625 *μ*M to 61% at 5 *μ*M PM (p<0.05). Similarly, the percentage of Annexin V-FITC-binding Panc-1 cells increased from 25% at 0.625 *μ*M to 57% at 5 *μ*M PM (p<0.05).

The induction of apoptosis by PM was confirmed by the cleavage of PARP-1 by western blot analysis. As shown in Fig. 2B, PARP-1 was cleaved in both cell lines at PM concentrations of 2.5 to 5 *μ*M.

### Pristimerin causes cleavage of procaspases in pancreatic cancer cells

**Figure 3.**
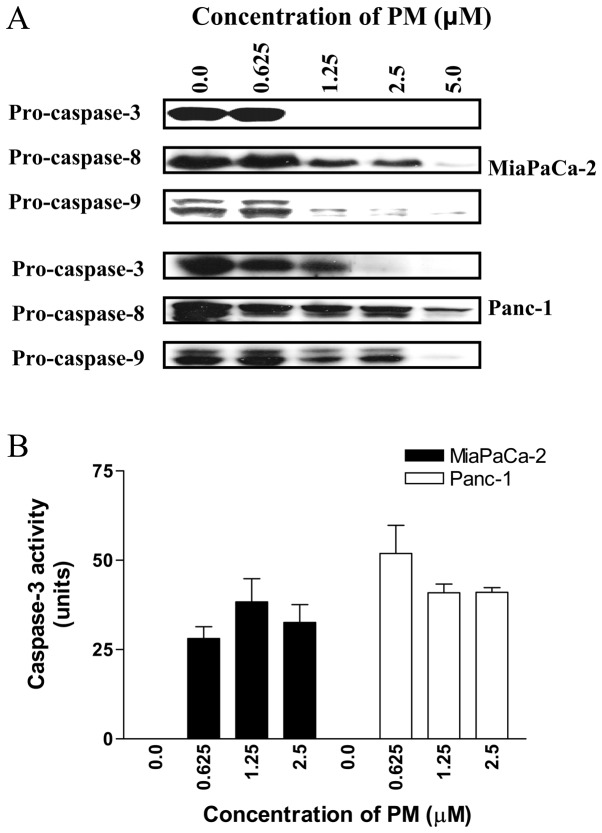
PM induces processing of procaspases. (A) Whole cell extracts prepared from MiaPaCa-2 and Panc-1 cells treated with PM (0–5 *μ*M) for 20 h were analyzed by western blot analysis for processing of procaspases-3, -8 and -9. (B) PM increases the activity of caspase-3. MiaPaCa-2 and Panc-1 cells were treated with PM (0–2.5 *μ*M) for 20 h and caspase-3 activity in cell extracts was measured by using caspase-3 activity kit.

For further evidence that PM induces apoptosis in pancreatic cancer cells we also examined the effect of PM on the activation of procaspases-3, -8 and -9. Western blot analysis of cell lysates prepared from cells treated with PM showed partial to complete processing of procaspases-3, -8 and -9 in MiaPaCa-2 cells at 2.5 to 5 *μ*M PM (Fig. 3A). In Panc-1 cells, significant to complete processing of procaspases-3, -8 and -9 was evident at PM concentrations of 1.25 to 5 *μ*M.

We also measured caspase-3 in cells treated with PM using ApoAlertR caspase-3 assay kit from Clontech Laboratories. MiaPaCa-2 and Panc-1 cells treated with PM (0 to 2.5 *μ*M) for 20 h were processed according to the manufacturer’s protocol for the colorimetric measurement of caspase-3 activity. As shown in Fig. 3B, PM significantly induced caspase-3 activity at concentrations of 0.625 to 2.5 *μ*M in both cell lines compared to control cells (p<0.05).

Taken together, increase in Annexin V-FITC-binding cells, cleavage of PARP-1 and procaspases-3, -8, -9 and increase in caspase-3 activity indicated induction of apoptosis by PM.

### Pristimerin induces mitochondrial depolarization and release of cytochrome c

**Figure 4.**
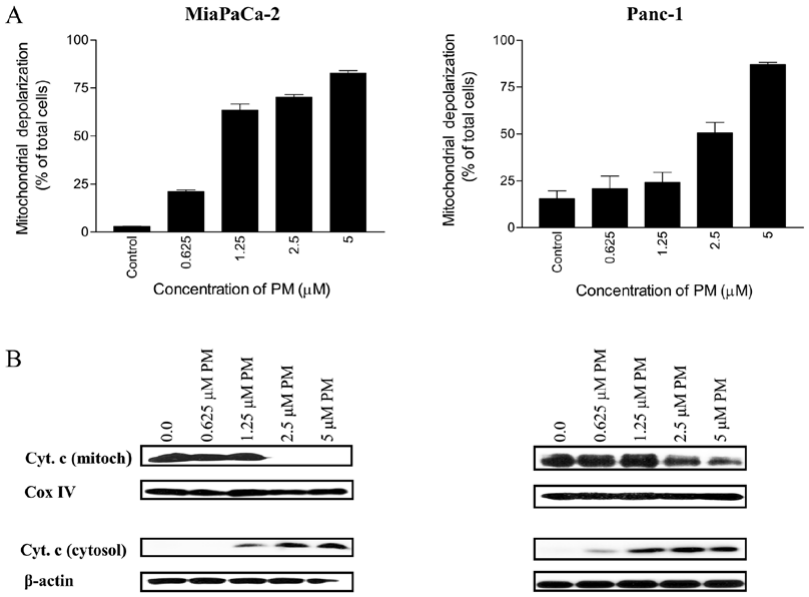
PM induces mitochondrial depolarization and release of cytochrome *c*. (A) MiaPaCa-2 and Panc-1 cells were treated with PM at 0 to 5 *μ*M for 20 h. Cells were loaded with mitochondrial potential sensor JC-1 (10 *μ*g/ml) for 10 min at 22°C and analyzed by flow cytometry for cells fluorescing red (FL2 channel) or green (FL1 channel). Data are presented as percentage of cells with loss of mitochondrial potential difference (mean ± S.D. of three independent experiments). ^*^p<0.05 compared to no PM controls. (B) Effect of PM on mitochondrial cytochrome *c*. After treatment with PM (0 to 5 *μ*M) for 20 h, mitochondrial and cytosolic fractions were prepared from using ApoAlert Cell Fractionation kit and cytochrome *c* was analyzed by western blot analysis. Data presented are from a representative experiment of three independent measurements.

Whether PM utilizes mitochondrial ‘intrinsic’ pathway in apoptotic death of pancreatic cancer cells was investigated next. For this, we evaluated the change in mitochondrial membrane potential following the treatment of cells with PM. Thus, MiaPaCa-2 and Panc-1 cells treated with PM (0.625–5 *μ*M) for 20 h were loaded with mitochondrial membrane-potential JC-1 probe and fluorescent shift in cells was measured by flow cytometry. As shown in Fig. 4A, the percentage of MiaPaCa-2 cells with green fluorescence changed from 3% at 0 *μ*M PM to 21, 63, 70 and 82% at 0.625, 1.25, 2.5 and 5 *μ*M PM, respectively. The percentage of Panc-1 cells with green fluorescence also increased significantly (p<0.05) after treatment with PM (e.g., 15, 21, 24, 50 and 87% of cells with green fluorescence at 0, 0.625, 1.25, 2.5 and 5 *μ*M PM, respectively).

To further determine the effect of PM on mitochondrial integrity, the release of cytochrome *c* from mitochondria in cells treated with PM was measured. Western blot analysis of mitochondrial and cytosolic fractions of MiaPaCa-2 and Panc-1 cells treated with PM (0.625–5 *μ*M) demonstrated the release of cytochrome *c* from the mitochondria in both cell lines (Fig. 4B). Mitochondrial cytochrome *c* levels were more than 90% decreased in MiaPaCa-2 cells at 2.5 *μ*M and were completely devoid of it at 5 *μ*M PM without significant change in Cox IV levels (loading control). PM also induced the release of cytochrome *c* from mitochondria in Panc-1 cells at 2.5 and 5 *μ*M PM. As expected, decrease in mitochondrial cytochrome *c* correlated with the corresponding increase in cytosolic cytochrome *c* in both cell lines (Fig. 4B). Together, these data demonstrated induction of mitochondrial ‘intrinsic’ pathway of apoptosis by PM in the pancreatic cancer cells.

### Pristimerin inhibits pro-survival signaling proteins in pancreatic cancer cells

**Figure 5.**
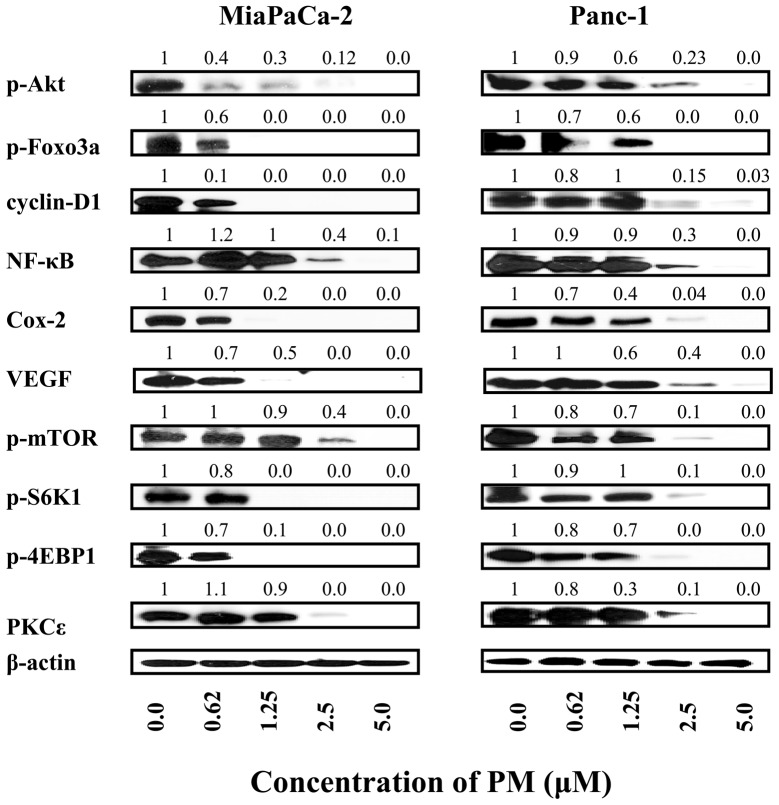
PM inhibits prosurvival signaling proteins Akt, NF-κB, mTOR and their downstream intermediaries. MiaPaCa-2 and Panc-1 cells were treated with PM at concentrations of 0 to 5 *μ*M for 20 h. After treatment, cell lysates were analyzed for p-Akt, NF-κB (p65) and mTOR as well as downstream targets of p-Akt (p-Foxo3a, cyclin D1), NF-κB (Cox-2, VEGF) and mTOR (p-S6K1, p-4E-BP1) by western blot analysis. Cell lysates were also analyzed for levels of PKCɛ. The uniformity of sample loading was determined by anti-β-actin antibody. Numbers above the lanes show relative change in signal strength compared to control. Each experiment was repeated three times.

Akt, NF-κB and mTOR are pro-survival signaling proteins that are constitutively active in a variety of human cancers and confer survival advantage and resistance of cancer cells to various forms of anticancer therapies. We investigated whether induction of apoptosis in pancreatic cancer cells by PM involved the inhibition of Akt, NF-κB, mTOR and downstream mediators of these signaling molecules. Cellular lysates prepared from MiaPaCa-2 and Panc-1 cells treated with PM (0 to 5 *μ*M) for 20 h were analyzed by western blot analysis for levels of p-Akt and Akt regulated Foxo-3α and cyclin D1; NF-κB (p65) and NF-κB-regulated Cox-2 and VEGF; p-mTOR and mTOR-regulated p-S6K1 and p-4E-BP1. p-Akt, NF-κB and p-mTOR were significantly to completely inhibited in both cell lines by PM at concentrations of 1.25 to 5 *μ*M (Fig. 5). PM also inhibited the levels of various downstream mediators of these signaling proteins at similar concentrations. Since PKCɛ has been invoked in induction of apoptosis by anticancer agents we analyzed the effect of PM on the levels of PKCɛ. PM dramatically reduced the level of PKCɛ in both cell lines at concentrations of 2.5 to 5 *μ*M, suggesting that PKCɛ-mediated apoptotic pathway is also a target of PM. Together, these data indicated that induction of apoptosis in pancreatic cancer cells by PM involves the inhibition of pro-survival p-Akt, NF-κB and mTOR and their downstream mediators as well as PKCɛ mediated pathway of apoptosis.

### Pristimerin downregulates anti-apoptotic Bcl-2 but not Bcl-xL expression in pancreatic cancer cells

**Figure 6.**
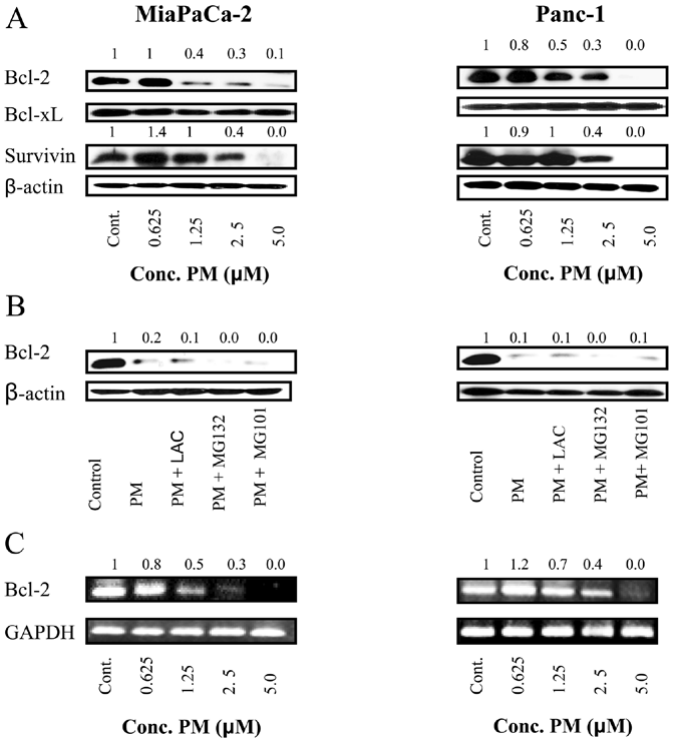
PM downregulates antiapoptotic Bcl-2 by inhibiting gene expression. (A) Western blot analysis of cell extracts of MiaPaCa-2 and Panc-1 cells treated with PM (0 to 5 *μ*M) for 20 h showing downregulation of Bcl-2 and survivin but not Bcl-xL. (B) Treatment of MiaPaCa-2 and Panc-1 cells with PM in the presence of lactacystin or MG132 (proteasomal inhibitors) or MG 101 (calpain inhibitor) does not prevent downregulation of Bcl-2 by PM. (C) PM inhibits Bcl-2 mRNA expression in MiaPaCa-2 and Panc-1 cells as analyzed by RT-PCR. Numbers above the lanes show relative change in signal strength compared to control. Each experiment was repeated two times.

Bcl-2 and Bcl-xL are anti-apoptotic proteins located in mitochondrial wall that regulate mitochondrial or ‘intrinsic’ apoptosis pathway by controlling mitochondrial permeability. We determined the effect of PM on expression of these proteins in MiaPaCa-2 and Panc-1 cells. Lysates of cells treated with PM (0.625 to 5 *μ*M) for 20 h were analyzed by western blot analysis for the levels of Bcl-2 and Bcl-xL. As shown in Fig. 6A, PM partially to completely inhibited Bcl-2 in both cell lines at 1.25 to 5 *μ*M PM. In contrast, the levels of Bcl-xL were not affected by PM in these cell lines. Furthermore, survivin, which is an inhibitor of apoptosis was also reduced by PM in both cell lines.

In order to investigate the mechanism by which PM inhibits Bcl-2, we examined whether PM mediates proteasomal or lysosomal degradation of Bcl-2. MiaPaCa-2 and Panc-1 cells were treated with PM (5 *μ*M) in the absence or presence of proteasomal inhibitors lactacystin and MG132 or calpain inhibitor MG101 for 20 h and cell lysates were analyzed for Bcl-2 by western blot analysis. As shown in Fig. 6B, neither proteasomal nor calpain inhibitors prevented the down-regulation of Bcl-2 by PM, indicating that PM does not induce proteolytic degradation of Bcl-2.

Next we evaluated the effect of PM on Bcl-2 gene expression by RT-PCR. Treatment with PM at 1.25 to 5 *μ*M for 20 h inhibited Bcl-2 mRNA in both cell lines in a concentration-related manner without affecting the expression of GAPDH, a housekeeping gene (Fig. 6C). Thus, RT-PCR data suggested that PM downregulates Bcl-2 by inhibiting Bcl-2 gene transcription.

## Discussion

Pristimerin containing plant products have been used in traditional medicine to treat a variety of diseases including inflammation and cancer. However, the purported medicinal effects of pristimerin have only recently come under scientific scrutiny and the antiproliferative and apoptosis-inducing activity of pristimerin has been validated against diverse types of tumor cell lines in a small number of studies (11–18). Although, these studies have provided some insights into the mode of cell death by pristimerin; the molecular mechanisms of the proapoptotic activity of pristimerin remain to be understood. In the present study, we investigated the mechanism of the antitumor activity of pristimerin in MiaPaCa-2 and Panc-1 pancreatic cancer cell lines with a focus on the effect of pristimerin on pro-survival (anti-apoptotic) cellular mechanisms. PM significantly inhibited the proliferation of both cell lines at concentrations of 1.25 to 5 *μ*M. The inhibition of cell proliferation by PM was associated with induction of apoptosis as demonstrated by the increased binding of Annexin V due to the externalization of phosphatidylserine to the outer leaflet of the cell membrane and cleavage of PARP-1, both well recognized markers of apoptosis. These results indicated that induction of apoptosis is part of the mechanism by which PM destroys pancreatic cancer cells and corroborate the result of a previous study that also showed induction of apoptosis in pancreatic cancer cells by PM (21).

Two major pathways of apoptotic cell death program have been identified, namely receptor-mediated (extrinsic) and mitochondrial (intrinsic) apoptotic cell death pathways (22). In both cases, caspases, a family of cysteine proteases, play an important role in the apoptotic cell death. In the extrinsic pathway, binding of the death ligands (e.g., TNF-α, FasL, TRAIL) with their cognate receptors activates initiator caspase-8 which then cleaves and activates effector caspases -3, -6 and -7 leading to apoptosis (23). In mitochondrial or intrinsic pathway, undefined signals induce release of cytochrome *c* from mitochondria, which in conjunction with Apaf-1 causes the activation of initiator caspase-9. Activated caspase-9, in turn, activates effector caspases-3, -6 and -7 (22). Pristimerin induced the cleavage of initiator procaspases-8 and -9 and effector caspase-3. The cleavage of procaspase-9 indicated that PM activates the mitochondrial (intrinsic) pathway of apoptosis in pancreatic cancer cells. The activation of the mitochondrial pathway by PM is also supported by the loss of mitochondrial membrane potential and release of cytochrome *c* from mitochondria in cancer cells treated with PM (Fig. 4). Thus, the cleavage of procaspase-9 and release of cytochrome *c* from mitochondria leading to the activation of effector caspase-3 indicated that the mitochondrial apoptosis pathway plays a significant role in the apoptotic cell death of pancreatic cancer cells by PM. The processing of procaspase-8 by PM suggested that the extrinsic pathway of apoptosis may also be involved in the apoptotic cell death of pancreatic cancer cells by PM. Activated caspase-8 may directly cleave and activate caspase-3 or it may facilitate caspase-9 mediated apoptosis through the truncation of Bid, a proapoptotic BH3 only Bcl-2 family member that links extrinsic pathway with the intrinsic pathway of apoptosis (24,25).

Phosphatidylinositol-3 kinase/Akt (PI3K/Akt)) signal transduction pathway which controls cell proliferation, survival, apoptosis and malignant transformation is frequently activated in a variety of malignancies including pancreatic cancer (26). p-Akt promotes cell growth and survival by inactivating downstream substrates such as Bad, procaspase-9, and forkhead transcription factors (27,28). Anti-apoptotic NF-κB and progrowth mTOR signaling pathways are downstream targets of activated Akt. NF-κB family of transcription factors controls the expression of genes involved in immune and inflammatory responses, cell proliferation, oncogenesis, angiogenesis and Bcl-2 family members (29). NF-κB plays a critical role in resistance of cancer cells to anticancer therapies by protecting them from apoptosis (30). mTOR is a 290 kDa serine-threonine kinase, which controls cell growth, survival, division and motility is activated in human tumors (31,32). PM inhibited p-Akt and its downstream targets Foxo-3α and cyclin D1; NF-κB (p65) and NF-κB-regulated Cox-2 and VEGF; p-mTOR and mTOR-regulated p-S6K1 and p-4E-BP1 in both pancreatic cancer cell lines. Thus, each of the three major pro-survival (anti-apoptotic) signaling pathways related proteins were inhibited by PM, suggesting that targeting multiple pro-survival signaling pathways may be critical for the antiproliferative and apoptosis-inducing activity of PM.

The intrinsic (mitochondrial) pathway of apoptosis is regulated by members of the Bcl-2 family of proteins and inhibitors of apoptosis (33,34). Bcl-2 and Bcl-xL are major anti-apoptotic members of Bcl-2 family that inhibit apoptosis by preventing the activation of inner mitochondrial permeability transition pore and release of proapotogenic mitochondrial contents including cytochrome *c* (33). PM inhibited Bcl-2 but had no effect on the expression of Bcl-xL. In addition to Bcl-2, PM also reduced the levels of anti-apoptic survivin. Thus, inhibition of these anti-apoptotic proteins seems essential for induction of apoptosis by PM. These data also imply that inhibition of Bcl-2 by PM is sufficient to facilitate Bax and Bak mediated mitochondrial permeability transition and release of cytochrome *c* without the inhibition of Bcl-xL.

The stability of Bcl-2 is regulated through post-translational modifications, such as dephosphorylation, ubiquitination and proteasomal degradation (35). However, PM-induced downregulation of Bcl-2 was not mediated through the proteolytic degradation, since treatment of tumor cells with PM in the presence of proteasomal and calpain inhibitors failed to prevent downregulation of Bcl-2. On the other hand, RT-PCR analysis revealed that the inhibition of Bcl-2 by PM is mediated through the inhibition of Bcl-2 gene transcription. Collectively, results of the present study suggest that a better understanding of the mechanism of the proapoptotic activity of PM can potentially facilitate the clinical development of pristimerin for the treatment of pancreatic cancer.
